# Supplementary material for: Reproducibility of knee extensor and flexor contraction velocity in healthy men and women assessed using tensiomyography: A registered report
Source: PLoS One. 2023 Aug 2;18(8):e0288806. doi: 10.1371/journal.pone.0288806 (PMC10395843; doi:10.1371/journal.pone.0288806)
Supplement: S1 Checklist — (PDF) [file pone.0288806.s001.pdf]

STROBE Statement regarding the manuscript titled “Reproducibility of knee extensor and flexor contraction velocity in healthy men and women assessed using tensiomyography: A registered report”

| | Item No. | Recommendation | Page No. | Relevant text from manuscript |
|---|---|---|---|---|
| **Title and abstract** | 1 | (*a*) Indicate the study’s design with a commonly used term in the title or the abstract | 1 | “Reproducibility of knee extensor and flexor contraction velocity in healthy men and women assessed using tensiomyography: A registered report” |
| | | (*b*) Provide in the abstract an informative and balanced summary of what was done and what was found | 2-3 | “[…] Methods<br>On two consecutive days, we determined Vc of the biceps femoris and rectus femoris of twenty-four healthy subjects. The maximum displacement was determined twice within three minutes on day one and a third time 24 h later. Also, on day two, we applied three blocks of ten consecutive stimuli at a constant intensity of 50 mA, separated by 3 min each. Inter-stimuli intervals in randomly ordered blocks were 10 s, 20 s or 30 s, respectively.<br>Results<br>All Vc concepts displayed good to excellent relative (ICC 0.87 – 0.99) and generally good absolute within- and between-day reliability for both muscles. Across Vc-concepts, absolute reliability was higher for the rectus femoris (CV% 1.3 – 7.95 %) compared to the biceps femoris (CV% 6.06 - 15.30 %). In both muscles, Vc was generally not affected by different inter-stimuli intervals. For most Vc concepts, repeated stimulation induced an increase regardless of the inter-stimuli interval, but this effect was mainly trivial and small at most. […]” |
| **Introduction** | | | | |
| Background/rationale | 2 | Explain the scientific background and rationale for the investigation being reported | 3-5 | “[…] Tensiomyography (TMG) measures the radial displacement of a muscle belly during an electrically stimulated twitch response. […] an increasing number of studies reports the rate of displacement (Vc), represented by the slope of the radial displacement curve. Several different concepts exist to calculate Vc but there is no consensus on the most suitable formula [27]. Further, there is a lack of studies investigating the reproducibility of these concepts, […] variations within current methodical approaches exist regarding the electrical stimulation procedure to determine the radial displacement curve from which Vc |

| | | | | |
|---|---|---|---|---|
| | | | | is calculated. […] previous studies mainly used an inter-stimulus interval (ISI) of 10 s or 15 s to avoid fatigue or post-tetanic potentiation. However, they also stated a lack of studies investigating the optimal rest interval between consecutive stimuli [1]. […] studies mentioned above did not report Vc, so the effect of ISI during repeated stimulation on Vc is still unclear.” |
| Objectives | 3 | State specific objectives, including any prespecified hypotheses | 5 | “[…] the first aim of this study was to investigate the within and between-day reliability of the five most frequently applied concepts to calculate Vc. The second aim was to investigate the effect of different ISI during repeated stimulation on Vc, assessed by the example of biceps femoris and rectus femoris. Finally, we hypothesized that Vc would be affected by changing ISI during ten repeated stimuli at a constant stimulation intensity.” |
| **Methods** | | | | |
| Study design | 4 | Present key elements of study design early in the paper | 5, 6 | “This study was a single group reliability study with repeated measurements within two consecutive days. […] We determined the absolute and relative within- and between-day reliability of the five most frequently used concepts to determine Vc of the BF and RF as assessed via TMG. We also determined the effect of three different rest intervals between ten consecutive stimuli at a constant intensity on these Vc concepts.” |
| Setting | 5 | Describe the setting, locations, and relevant dates, including periods of recruitment, exposure, follow-up, and data collection | 6 | “Participants were recruited at the Institute for Applied Training Science and the Sport Sciences Department of the University of Leipzig from early Mai to the end of June 2022. During the same period, all data were collected at the Institute for Applied Trainings Science in Leipzig.” |
| Participants | 6 | (*a*) *Cohort study*—Give the eligibility criteria, and the sources and methods of selection of participants. Describe methods of follow-up<br>*Case-control study*—Give the eligibility criteria, and the sources and methods of case ascertainment and control selection. Give the rationale for the choice of cases and controls | 6 | referring to (a):<br>“We included 24 women and men in this study who were required to meet the following inclusion criteria: healthy, aged between 18-40 years, physically active for a minimum of three times per<br>week. According to the World Health Organization, physical activity was defined as any body movement produced by skeletal muscles, including activities related to transportation, leisure time or work [36]. Exclusion criteria were the following: pregnancy, history of neuromuscular or musculoskeletal disorders, pain, or injury in the lower limbs during the last six months, previous surgical treatment to the lower limbs, practising sport on a professional level, taking prescribed medication, nontolerance or any |

| | | | | |
|---|---|---|---|---|
| | | *Cross-sectional study*—Give the eligibility criteria, and the sources and methods of selection of participants | | contraindication to electrical stimulation using self-adhesive electrodes, and wearing an implanted medical device.” |
| | | (*b*) *Cohort study*—For matched studies, give matching criteria and number of exposed and unexposed<br>*Case-control study*—For matched studies, give matching criteria and the number of controls per case | n.a. | |
| Variables | 7 | Clearly define all outcomes, exposures, predictors, potential confounders, and effect modifiers. Give diagnostic criteria, if applicable | 10-11 | “The TMG software automatically calculated the following parameters (Fig 2A): maximum radial displacement (Dm, mm), delay time, which refers to the time interval between the stimulus and 10% of Dm (Td, ms) and contraction time, which refers to the time interval between 10% and 90% of Dm (Tc, ms). From these data, we determined Vc according to the five most frequently used concepts [27], as shown in Table 2.” |
| Data sources/ measurement | 8* | For each variable of interest, give sources of data and details of methods of assessment (measurement). Describe comparability of assessment methods if there is more than one group | 8-10 | “To perform tensiomyographic measurements, we used a TMG-S1 electrical stimulator (TMG-BMC d.o.o., Ljubljana, Slovenia), a GD30 displacement sensor (Panoptik d.o.o., Ljubljana, Slovenia) and two squared self-adhesive electrodes (50x50 mm, Axion GmbH, Leonberg, Germany). The signal of the displacement sensor was recorded using the TMG Software v3.6 (TMG-BMC d.o.o., Ljubljana, Slovenia).<br>All measurements were performed on the BF and RF of the dominant leg. […]”<br>all further Details of how the measurements were performed are reported on pages 8-10 in section “Experimental set-up and procedures” |
| Bias | 9 | Describe any efforts to address potential sources of bias | 7-8 | “On the first day, we familiarized all participants with the electrical stimulation procedure by applying two stimuli with a duration of 1 ms each at 20 mA and 30 mA to the RF, followed by a three-minute rest before starting the actual measurement [30]. All measurements were taken at the same time of the day, and a constant room temperature of 21 ± 1 °C. According to a recent study by Domaszewski et al., orally administered caffeine can affect contractile parameters assessed by TMG [38]. Consequently, participants were asked to refrain from caffeine intake for 2 h preceding all measurements and to avoid alcohol consumption and fatiguing exercise for 24 h before the start and during the trial to counteract possible confounding. Further, to prevent potential |

| | | | | |
|---|---|---|---|---|
| | | | | confounding by variations in hydration [39] and in line with a previous study [26], participants were asked to record their total dietary intake during the 24 h before the first appointment and replicate their intake during the 24 h before the second visit (S1 Appendix). […]<br>We used the online application RESEARCH RANDOMIZER [40] to conduct a block randomization procedure [41] to determine the order of ISI during repeated stimulations for each participant. The randomisation procedure was based on four blocks corresponding to the number of possible orders of the three different ISI. Each block contained six different numbers to account for a total of 24 subjects. To blind the rater from results of previous measurements, we chose the settings of the TMG measurement software as not to display prior measurements." |
| Study size | 10 | Explain how the study size was arrived at | 8 | "Two different calculations were made to justify the sample size for this study, which we described in detail in the study protocol of this registered report [35]. Shortly, as for our first aim to investigate the reproducibility of Vc concepts, assuming the lowest ICC reported of 0.92 [29], two measurements per subject, and an alpha error level of 0.05, 18 subjects were needed to achieve a desired precision of a confidence interval of 0.15.<br>As for our second aim to detect an effect of changing the interstimulus interval on Vc, our calculation was based on the raw data provided by Wilson et al. [30]. As we assumed three groups, ten measurements per subject, an effect size f = 3.1 (Vc10-90%) and f = 1.6 ($Vc_{norm}$), a nonsphericity correction coefficient of 0.7 (Vc10-90%) and 0.6 ($Vc_{norm}$), an alpha error level of 0.01, a power of 0.95, a number of 9 (Vc10-90%) subjects or 18 ($Vc_{norm}$) subjects were needed, respectively. We confirmed the estimated sample size for the ANOVA by repeating the calculations for both Vc10-90% and $Vc_{norm}$ using g*power v3.1.9.2 [42]. Based on these results and accounting for potential dropouts, we recruited 24 subjects." |
| Quantitative variables | 11 | Explain how quantitative variables were handled in the analyses. If applicable, describe which groupings were chosen and why | 10 | "From M1, M2 and M3, the two displacement curves with the highest first peak of the radial displacement curve of each measurement were averaged, respectively, and used for further analysis. From block 1, block 2 and block 3, we used every single displacement curve for further analysis." |

| | | | | |
|---|---|---|---|---|
| Statistical methods | 12 | (*a*) Describe all statistical methods, including those used to control for confounding | 11-13 | All statistical methods are detailed on pages 11-13.<br>In addition to that, the code used to perform these analyses will be openly available after publication, as indicated on page 13. |
| | | (*b*) Describe any methods used to examine subgroups and interactions | 11-13 | see above |
| | | (*c*) Explain how missing data were addressed | 7 | "*Two measurements were removed from the data for all analyses regarding M1 and M2 by listwise deletion. In both cases, Dm was unexpectedly identified as a second higher peak than the first peak of the respective displacement curve despite similar shapes of the displacement curves and first peak's amplitudes at both time points (M1 and M2). This led to outliers in the difference scores between M1 - M2 of several variables." |
| | | (*d*) *Cohort study*—If applicable, explain how loss to follow-up was addressed<br>*Case-control study*—If applicable, explain how matching of cases and controls was addressed<br>*Cross-sectional study*—If applicable, describe analytical methods taking account of sampling strategy | n.a. | |
| | | (*e*) Describe any sensitivity analyses | n.a. | |
| **Results** | | | | |
| Participants | 13* | (a) Report numbers of individuals at each stage of study—eg numbers potentially eligible, examined for eligibility, confirmed eligible, included in the study, completing follow-up, and analysed | 13 | "The measurement protocol was well tolerated by the subjects and all 24 subjects fully completed the study." |
| | | (b) Give reasons for non-participation at each stage | 7 | shown in Fig 1 on page 7<br>"*Two measurements were removed from the data for all analyses regarding M1 and M2 by listwise deletion. In both cases, Dm was unexpectedly identified as a second higher peak than the first peak of the respective displacement curve despite similar shapes of the displacement curves and first peak's amplitudes at both time points (M1 and M2). This led to outliers in the difference scores between M1 M2 of several variables." |

| | | | | |
|---|---|---|---|---|
| | | (c) Consider use of a flow diagram | 7 | Fig 1 shows the study flow and the number of measurements resp. participants included in the analyses |
| Descriptive data | 14* | (a) Give characteristics of study participants (eg demographic, clinical, social) and information on exposures and potential confounders | 6 | Table 1 shows the anthropometric data and measures of the physical activity level of all included participants |
| | | (b) Indicate number of participants with missing data for each variable of interest | 7 | shown in Fig 1 on page 7<br>"*Two measurements were removed from the data for all analyses regarding M1 and M1 by listwise deletion. In both cases, Dm was unexpectedly identified as a second higher peak than the first peak of the respective displacement curve despite similar shapes of the displacement curves and first peak's amplitudes at both time points (M1 and M2). This led to outliers in the difference scores between M1 - M2 of several variables." |
| | | (c) *Cohort study*—Summarise follow-up time (eg, average and total amount) | n.a. | |
| Outcome data | 15* | *Cohort study*—Report numbers of outcome events or summary measures over time | *n.a.* | |
| | | *Case-control study*—Report numbers in each exposure category, or summary measures of exposure | *n.a.* | |
| | | *Cross-sectional study*—Report numbers of outcome events or summary measures | 14, 17, 19 | see Tables 3, 4 and 5 |
| Main results | 16 | (*a*) Give unadjusted estimates and, if applicable, confounder-adjusted estimates and their precision (eg, 95% confidence interval). Make clear which confounders were adjusted for and why they were included | n.a. | |
| | | (*b*) Report category boundaries when continuous variables were categorized | n.a. | |

| | | | | |
|---|---|---|---|---|
| | | (*c*) If relevant, consider translating estimates of relative risk into absolute risk for a meaningful time period | n.a. | |
| Other analyses | 17 | Report other analyses done—eg analyses of subgroups and interactions, and sensitivity analyses | n.a. | |
| **Discussion** | | | | |
| Key results | 18 | Summarise key results with reference to study objectives | 22 | "This study compared the five most frequently used concepts to determine Vc in terms of within and between-day reliability, evaluated in the biceps femoris muscle and rectus femoris muscle. The relative within- and between-day reliability of all Vc concepts was good to excellent for both the BF and RF. The absolute reliability was adequate for the RF within and between days across all Vc concepts, as assessed by the CV%. For the BF, Vc concepts exhibited sufficient within-day reliability but insufficient between-day reliability (i.e., CV% above 10 %) in three of five concepts.<br>Further, this study investigated whether Vc would be affected by rest intervals of 10 s, 20 s or 30 s during ten consecutive stimuli at a constant intensity of 50 mA. Contrary to our initial hypothesis, ISI did not affect Vc of the BF or RF during repeated stimulation. However, repeated stimulation significantly affected the majority of Vc concepts regardless of ISI in both muscles, but these effects were trivial to small. This is the first study to investigate the effect of different ISI during repeated stimulation on the most frequently used Vc concepts." |
| Limitations | 19 | Discuss limitations of the study, taking into account sources of potential bias or imprecision. Discuss both direction and magnitude of any potential bias | 25 | "A limitation of our study is that we only assessed two muscles of the lower extremities. Nevertheless, as our results show, the absolute reliability of Vc appears to be muscle specific. Therefore, we suggest that future studies should investigate the reproducibility of Vc in a broader range of muscles, including muscles of the upper extremities. Another limitation of our study is that we included only healthy and physically active women and men between 18 and 40 years of age. The effect of different ISI on Vc might differ in older subjects or athletes due to age- and training-related shifts in the muscle fibre spectrum [11,65,66]." |
| Interpretation | 20 | Give a cautious overall interpretation of results considering objectives, limitations, | 22-23, 25 | "Concerning the muscles investigated in this study, previous studies reported a good to excellent relative reliability (ICC scores from 0.82 to 0.99 for Td, Tc, Dm) [29,56,59,60] |

| | | | | |
|---|---|---|---|---|
| | | multiplicity of analyses, results from similar studies, and other relevant evidence | | for the BF but only partially sufficient absolute reliability (CV% scores from 2.4 % to 19.8 % for Td, Tc, Dm) [29,56,60]. To the RF, ICC scores from 0.87 to 0.92 and CV% scores from 3.8 % to 9.3 % have been reported [29], thus representing good to excellent relative and sufficient absolute reliability of Td, Tc and Dm. Accordingly, our results (ICC scores from 0.90 to 0.99 and CV% scores from 0.42 % to 9.30 %) are consistent with previous findings, demonstrating excellent relative and acceptable absolute within- and between-day reliability for Td, Tc and Dm of the BF and RF, respectively. […] Thus, although repeated stimulation with an ISI of 10 s, 20 s or 30 s may induce a potentiation effect on Vc and generic TMG parameters, the magnitude of this effect appears to be small at most. Consequently, in line with Latella et al. [31], our results suggest that applying a longer ISI of 30 s or 20 s instead of 10 s may provide no benefit in terms of methodical validity. In contrast, an ISI of 10 s may be preferable to a longer ISI in favour of a reduced time per measurement." |
| Generalisability | 21 | Discuss the generalisability (external validity) of the study results | 25-26 | "The five most frequently used Vc concepts displayed good to excellent relative and generally acceptable absolute within- and between-day reliability, as assessed for the BF and RF. $Vc_{norm}$ represented the highest overall reliability across time points and muscles within Vc concepts. However, we found muscle-specific differences in measures of absolute reliability, which may be attributable to anatomical differences between BF and RF. These results suggest that the reproducibility of Vc concepts needs further investigation in different muscles. Further, Vc and generic TMG parameters were generally not affected by different ISI during repeated submaximal stimulation. Repeated stimulation induced a potentiation effect regardless of ISI, resulting in an increase in Vc in most Vc concepts. However, the magnitude of this effect was mainly trivial and small at most. Consequently, according to our results, an ISI of 10 s might be preferable to a longer ISI in favour of less time required per measurement. However, as this is the first study to report the effect of different ISI on Vc during repeated stimulation, we suggest that this effect be further investigated in future studies." |
| **Other information** | | | | |
| Funding | 22 | Give the source of funding and the role of the funders for the present study and, if | n.a. | The authors received no funding. |

| | | |
|---|---|---|
| | applicable, for the original study on which the present article is based | |

*Give information separately for cases and controls in case-control studies and, if applicable, for exposed and unexposed groups in cohort and cross-sectional studies.

**Note:** An Explanation and Elaboration article discusses each checklist item and gives methodological background and published examples of transparent reporting. The STROBE checklist is best used in conjunction with this article (freely available on the Web sites of PLoS Medicine at http://www.plosmedicine.org/, Annals of Internal Medicine at http://www.annals.org/, and Epidemiology at http://www.epidem.com/). Information on the STROBE Initiative is available at www.strobe-statement.org.
