# Supplementary material for: Reproducibility of knee extensor and flexor contraction velocity in healthy men and women assessed using tensiomyography: A registered report
Source: PLoS One. 2023 Aug 2;18(8):e0288806. doi: 10.1371/journal.pone.0288806 (PMC10395843; doi:10.1371/journal.pone.0288806)
Supplement: S1 Appendix — (PDF) [file pone.0288806.s002.pdf]

# My food diary

This template was adapted from the German Society for Nutrition e. V.
The original German version of the diary is openly available via the following download link: https://www.dge-medienservice.de/mein-ernahrungstagebuch.html

Name:

Time period:

Goal:

**Your food diary - instructions on how to fill it in**

A food diary helps you to identify habits. Take the time to write down what and how much you eat and drink over one week. After this week, reflect: Are you eating enough vegetables and fruit? Are you drinking enough? Do you spend a lot of time eating in front of the TV or eat while you're doing something else? What do you find easy, what can you improve?

A nutritionist can help you with the evaluation. You can find addresses of qualified nutritionists under "Service" on the website www.dge.de.

This is how you use the diet diary:

- On the cover sheet, state your name and the period during which you will complete the food diary. Set a goal for this period.
- Fill in one sheet every day. If you need more space, use the flipside or another sheet.
- Write down everything you eat and drink - during a meal and also snacks.
- Enter your details directly after eating, so that you don't forget anything.
- Describe everything as precisely as possible. For example, include the fat content of dairy products.
- If you do not know the weight of the food or if you cannot weigh it, estimate the amount as well as possible, e.g. 1 teaspoon, 1 slice, 1 palm size.
- For drinks, also indicate the type, e.g. mineral water, tap water, black tea, fruit juice.
- Write down if there were any particularities, e.g. discomfort after eating, time pressure while eating, snacking in the car. You can also enter any medication you are taking here. Use the column "Notes/Situation" for this purpose.
- In the column "Activities" you can enter information about your exercise. Everything counts here!
- So don't just enter your sporting activities, but also note if you use the stairs instead of the lift or go for a walk during your lunch break.
- Under "Notes" you can write down everything that seems important to you. Perhaps you would like to write down a daily motto. There is also space here for special details, e.g. working day or weekend, day of holiday or illness, when you got up and went to bed or how you felt that day.

| Time<br>When? | Meal/Quantity<br>What?/How much? | Beverages/Quantity?<br>What?/How much? | Notes/Situation<br>How did I feel? | Activities<br>Which physical activities have I done? |
|---|---|---|---|---|
| 7:00 a.m. | 2 slices wholemeal bread, 2 tsp. butter, 1 tsp. jam, 1 slice Gouda 45 % F.i.Tr. | 1 cup coffee (125 ml) 4 tsp milk | a little in a hurry, at home | cycling to work |
| 10.30 a.m. | 2 large carrots (150 g), 1 chewing gum | 1 glass of mineral water (250 ml) | Carrots tasted good, office | Took the stairs instead of the lift! |
| 1.30 p.m. | 1 Wiener Schnitzel (2 palm sizes), breaded and deep-fried, 2 ladles of potato salad | 1 glass of mineral water (400 ml) | eaten frantically because of meeting, canteen | |
| 2.00 p.m. | 3 chocolate biscuits | 2 cups coffee (125 ml), 2 sachets coffee milk light, 4 tsp sugar, 1 small bottle mineral water (250 ml) | meeting, unpleasant gut feeling | |
| … | | | | |

My food diary

Name Date

| Time<br>When? | Meal/Quantity<br>What?/How much? | Beverages/Quantity?<br>What?/How much? | Notes/Situation<br>How did I feel? | Activities<br>Which physical activities have I done? |
|---|---|---|---|---|
| | | | | |
| | | | | |
| | | | | |
| | | | | |
| | | | | |
| | | | | |
| | | | | |
| | | | | |
| | | | | |
| | | | | |
| | | | | |
| | | | | |
| | | | | |
| | | | | |
| | | | | |
| | | | | |
| | | | | |

| Notes | |
|---|---|
| | |
| | |

My food diary

Name Date

| **Time**<br>**When?** | **Meal/Quantity**<br>**What?/How much?** | **Beverages/Quantity?**<br>**What?/How much?** | **Notes/Situation**<br>**How did I feel?** | **Activities**<br>**Which physical activities have I done?** |
|---|---|---|---|---|
| | | | | |
| | | | | |
| | | | | |
| | | | | |
| | | | | |
| | | | | |
| | | | | |
| | | | | |
| | | | | |
| | | | | |
| | | | | |
| | | | | |
| | | | | |
| | | | | |
| | | | | |
| | | | | |
| | | | | |

| Notes | |
|---|---|
| | |
| | |

My food diary

Name Date

| **Time**<br>**When?** | **Meal/Quantity**<br>**What?/How much?** | **Beverages/Quantity?**<br>**What?/How much?** | **Notes/Situation**<br>**How did I feel?** | **Activities**<br>**Which physical activities have I done?** |
|---|---|---|---|---|
| | | | | |
| | | | | |
| | | | | |
| | | | | |
| | | | | |
| | | | | |
| | | | | |
| | | | | |
| | | | | |
| | | | | |
| | | | | |
| | | | | |
| | | | | |
| | | | | |
| | | | | |
| | | | | |
| | | | | |

| Notes | |
|---|---|
| | |
| | |
